# Label-free detection of transporter activity via GPCR signalling in living cells: A case for SLC29A1, the equilibrative nucleoside transporter 1

**DOI:** 10.1038/s41598-019-48829-3

**Published:** 2019-09-24

**Authors:** Anna Vlachodimou, Adriaan P. IJzerman, Laura H. Heitman

**Affiliations:** Division of Drug Discovery and Safety, Leiden Academic Centre for Drug Research (LACDR), Leiden University, P.O. Box 9502, 2300 RA Leiden, The Netherlands

**Keywords:** Membrane proteins, Extracellular signalling molecules, Phenotypic screening, Receptor pharmacology

## Abstract

Transporters are important therapeutic but yet understudied targets due to lack of available assays. Here we describe a novel label-free, whole-cell method for the functional assessment of Solute Carrier (SLC) inhibitors. As many SLC substrates are also ligands for G protein-coupled receptors (GPCRs), transporter inhibition may affect GPCR signalling due to a change in extracellular concentration of the substrate/ligand, which can be monitored by an impedance-based label-free assay. For this study, a prototypical SLC/GPCR pair was selected, i.e. the equilibrative nucleoside transporter-1 (SLC29A1/ENT1) and an adenosine receptor (AR), for which adenosine is the substrate/ligand. ENT1 inhibition with three reference compounds was monitored sensitively via AR activation on human osteosarcoma cells. Firstly, the inhibitor addition resulted in an increased apparent potency of adenosine. Secondly, all inhibitors concentration-dependently increased the extracellular adenosine concentration, resulting in an indirect quantitative assessment of their potencies. Additionally, AR activation was abolished by AR antagonists, confirming that the monitored impedance was AR-mediated. In summary, we developed a novel assay as an *in vitro* model system that reliably assessed the potency of SLC29A1 inhibitors via AR signalling. As such, the method may be applied broadly as it has the potential to study a multitude of SLCs via concomitant GPCR signalling.

## Introduction

Solute carriers (SLCs) are transmembrane transport proteins that control a cell’s intra- and extracellular communication with its environment by regulating the translocation of small molecules, inorganic ions or other proteins across biological membranes. Their function as gatekeepers, their ubiquitous presence in the human body, and the large number of SLCs (more than 400) render them potential therapeutic targets^1^. However, their in-depth investigation has been limited as not many cellular assays are available to study transporter activity, making drug discovery difficult. The main and most well-known assay format currently used is the uptake assay, which measures the accumulation of a radiolabeled substrate in cells expressing the transporter under investigation^2^. Such assay however, presents limitations as only the end-point of the assay can be measured and/or washing steps are needed, which make this type of assay laborious and prone to artefacts. Moreover, the need for radioactive substrates has disadvantages, such as high costs, handling of radioactivity and storage of radioactive waste^3^. In addition, label-free electrophysiological methods have been applied and many electrogenic SLCs, i.e. transporters the function of which results in a positive or negative intracellular net charge, have been studied as such (Grewer *et al*. 2013)^4^. The best-known electrophysiological technique is patch-clamp, a well-studied concept that measures the direct current generated by ions that flow through the transporters, with a plethora of instruments being commercially available (Priest *et al*. 2004)^5^. In addition to patch-clamp, solid supported membrane (SSM)-based electrophysiology has been applied to transporters as a more sensitive approach, where SURFE²R N1 (Nanion Technologies) is the main instrumentation used^6^. However, no label-free assays applicable to non-electrogenic membrane transporters, which represent the majority of SLCs are available. Therefore, the development of a homogenous, kinetic and label-free assay in the field of non-electrogenic membrane transporter research is of utmost interest.

Label-free whole cell assays typically use a biosensor that detects physical properties of cells, such as size, adhesion and morphology, in order to measure cellular responses upon ligand stimulation^7^. The main advantage of using biosensors and cell morphology to functionally assess a ligand-target interaction is that cells are monitored in real time and in high sensitivity, making it possible to study cell systems with endogenous target expression^8,9^. As a result, no modification of the cells, for example transfection of an (engineered) target into an artificial cell line, or the compounds, i.e. with a fluorescent or radioactive tag, is necessary, which avoids potential artefacts^10^. Over the last decade, the number of available label-free cellular assays has significantly increased, and many of these have been used to study G protein-coupled receptor (GPCR) signalling^11,12^. Examples are the EPIC (Corning Inc.) and xCELLigence (ACEA Biosciences), which measure cell morphology optically and by impedance, respectively. However, the use of label-free assays are still rather unexploited, if at all, for membrane transporters.

ENT1 (also known as SLC29A1) is the most abundant nucleoside transport (NT) protein in the human body^13^. This SLC plays a crucial role in the provision of nucleosides, as it participates in the salvage pathways of nucleotide synthesis in cells lacking *de novo* biosynthetic pathways^14^. Therapeutically, ENT1 can be targeted by drugs that are both substrates and inhibitors. In the cases of viral infections and in some types of cancer, ENT1 transports well-known drugs inside the cell in order to exert their action, e.g. gemcitabine and ribavirin, respectively^15,16^. As far as ENT1 inhibition is concerned, molecules that diminish ENT1 activity are proposed as an add-on treatment of cancer, whenever ENT1 is overexpressed^17^. Moreover, ENT1 inhibitors can potentially be used in the treatment of ischemic heart disease^18^, stroke^19^ and inflammatory diseases^20^. Of note, in many cases the therapeutic effect of ENT1 inhibitors is induced by adenosine^21^, as its increased extracellular concentration can potentiate neuroprotective and cardioprotective actions resulting from the activation of neighbouring adenosine receptors (ARs).

**Figure 1:**
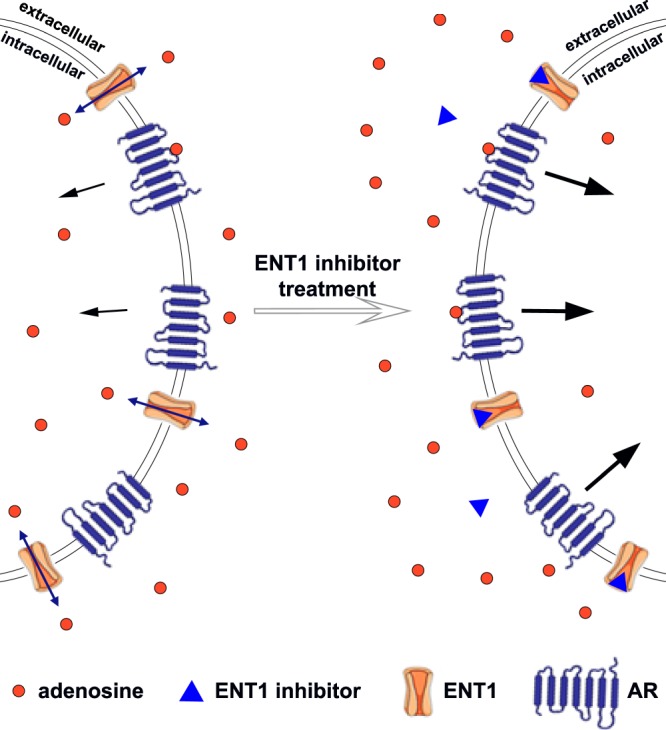
Assay principle. ENT1 equilibrates adenosine concentrations inside and outside of the cell membrane. Extracellular adenosine binds to ARs and causes their activation and signalling (black arrows). After treatment with an ENT1 inhibitor, adenosine cannot be translocated intracellularly with the same efficiency, depending on inhibitor’s inhibitory potency and concentration. The resulting higher extracellular adenosine concentration will cause increased AR activation (thicker arrows).

In the current study, we describe the development of a novel cellular assay for the functional assessment of SLC activity by using the label-free impedance-based xCELLigence instrument. Many endogenous substrates of membrane transporters are also ligands for GPCRs, e.g glutamate, dopamine and adenosine^22,23^. Thus, we hypothesized that by inhibiting a transporter, the substrate concentration would increase outside of the cell, resulting in increased GPCR signalling that is subsequently monitored with the xCELLigence (Fig. 1). For proof-of-principle, we investigated the inhibition of ENT1 transporters by well-known ENT1 inhibitors, and monitored concomitant adenosine receptor signalling. For validation purposes we performed radioligand binding studies on SLC29A1 as well.

## Results

### Assay development and optimization

To confirm the suitability of U-2 OS cells for studying ENT1 function via AR signalling, we performed a radioligand binding assay on U-2 OS membranes. U-2 OS membranes were incubated with [^3^H]NBTI and increasing concentrations of reference inhibitors, i.e. NBTI, dilazep and dipyridamole. All inhibitors fully displaced the radioligand from the ENT1. NBTI had the highest affinity (pK_i_ = 8.7 ± 0.02), followed by dilazep (pK_i_ = 8.5 ± 0.1) and dipyridamole (pK_i_ = 7.2 ± 0.1) (Supplementary Material; Fig. S1 and Table S1).

**Figure 2:**
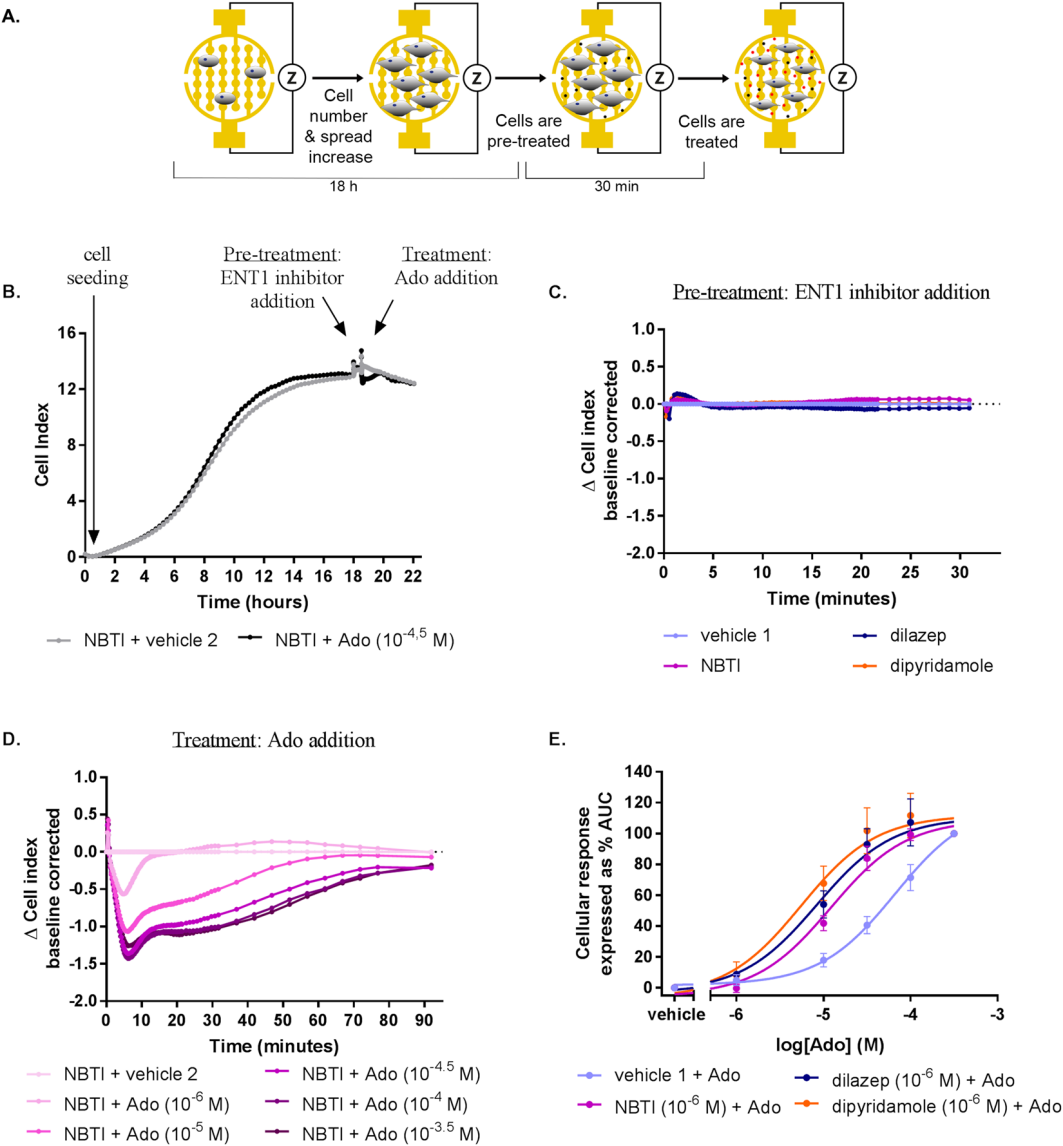
Concentration-dependent effects of adenosine (Ado) after cell pre-treatment with a single concentration of ENT1 inhibitors (“Format 1”). (**A**) Graphic representation of cell seeding, spreading, pre-treatment and treatment protocol. (**B**) Representative xCELLigence traces of a full experiment when cells were pre-treated with NBTI (10^−6^ M) and subsequently stimulated with adenosine. A representative response (**C**) after NBTI, dilazep, dipyridamole pre-treatment and (**D**) after adenosine treatment of cells pre-treated with NBTI. (**E**) Concentration-response curves for adenosine with or without ENT1 inhibitors pre-treatment (adenosine 10^−3.5^ M response as 100%). Data shown are mean ± SEM from at least three separate experiments performed in duplicate.

Subsequently, U-2 OS whole cells were used to monitor the inhibitors’ activity in the impedance-based label-free technology. U-2 OS cells adhered strongly to the bottom of the wells and thus the gold-coated electrodes of the E-plates, and therefore no additional coating was necessary to obtain a signal. Various concentrations of cells per well were tested in order to achieve a uniform cell monolayer (Supplementary Material; Fig. S2), which was the case for a concentration of 20,000 cells/well. After cell seeding, attachment, spreading and overnight proliferation, this concentration resulted in a cell index (CI) ranging from 10.0 to 12.0 (Fig. 2A,B). Thus, 20,000 cells/well was selected for all further experiments, as it allowed reliable and reproducible measurements of ENT1 inhibition and subsequent AR activation.

### Validation of the Assay Principle

For the development of the label-free assay, we hypothesized that ENT1 inhibition can be detected via adenosine receptor signalling, due to changes in the extracellular concentration of adenosine.

Firstly, it was examined if ENT1 inhibition would lead to an increased extracellular adenosine concentration. Hence, cells were pre-treated with a single concentration (10^−6^ M) of an ENT1 inhibitor and subsequently treated with several concentrations of adenosine (i.e. “Format 1”). Upon pre-treatment of cells for 30 min with a single concentration of an inhibitor (10^−6^ M) no change of impedance (expressed as ΔCl) was detected (Fig. 2C). On the contrary, subsequent treatment of cells with various concentrations of adenosine led to a decrease of ΔCl (Fig. 2D). Specifically, the signal decreased in a concentration-dependent manner reaching a minimum ranging from −0.5 to −1.5 ΔCl after approximately 8 to 10 min. The initial decrease in ΔCI was followed by a slow increase back to baseline within 90 min. From these impedance changes, the AUC was determined and a concentration-response curve was obtained providing apparent potency values of adenosine for AR signalling after pre-treatment with vehicle and NBTI, dilazep and dipyridamole, i.e. pEC_50_ values of 4.2 ± 0.1, 4.9 ± 0.1, 5.1 ± 0.1, 5.2 ± 0.1, respectively (Fig. 2E and Table 1).

**Table 1:** Potency of adenosine obtained with whole cell impedance-based experiments performed with U-2 OS cells after pre-treatment with different ENT1 inhibitors at 1 μM (“Format 1”), and potency (pIC_50_) and inhibitory efficacy of ENT1 inhibitors obtained with whole cell impedance-based experiments performed with U-2 OS cells followed by adenosine treatment at 10^−4.5^ M (“Format 2”).

	Format 1	Format 2	
	Adenosine pEC_50_ ± SEM^a^	pIC_50_ ± SEM (IC_50_ (nM))	Inhibitory efficacy^b^ (%) ± SEM
vehicle 1	4.2 ± 0.1	n.a.	100 ± 5.6
+NBTI	4.9 ± 0.1 ****	8.3 ± 0.3 (2.6)	219 ± 7.3****
+Dilazep	5.1 ± 0.1 ****	10.1 ± 0.1 (0.1)	286 ± 19****
+Dipyridamole	5.2 ± 0.1 ****	8.6 ± 0.5 (8.1)	525 ± 7.3****

Values are mean ± SEM of at least three separate experiments performed in duplicate.

^a^Significance compared to vehicle’s 1 pEC_50_ was tested using one-way ANOVA with Dunnett’s post-hoc test. ****p < 0.0001.

^b^Data are normalized to maximal response of adenosine (10^−4.5^ M) of vehicle 1 (100%). Significance compared to vehicle 1 was tested using one-way ANOVA with Dunnett’s post-hoc test. ****p < 0.0001.

n.a.: not applicable.

**Figure 3:**
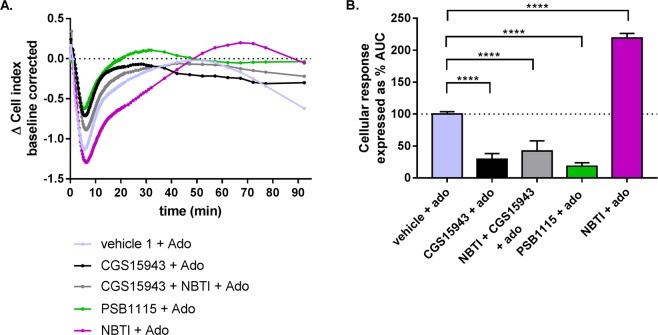
The response measured is adenosine receptor AR-mediated. (**A**) A representative response of adenosine (10^−4.5^ M) after pre-treatment with 10^−6^ M NBTI, CGS15943, PSB1115 or NBTI and CGS15943 simultaneously. (**B**) Bar graphs represent the AUC after adenosine addition (normalized to vehicle 1 as 100%). Data shown are mean ± SEM from at least three separate experiments performed in duplicate. Significance compared to vehicle was tested using one-way ANOVA with Tukey’s multiple comparison post-test. ****p < 0.0001.

Next, to confirm that the observed changes in impedance and the pEC_50_ values of adenosine are AR-specific, we used CGS 15943 as a non-selective AR antagonist and PSB 1115 as a selective A_2B_ AR antagonist. Cells were pre-treated with either CGS 15943, NBTI and PSB 1115 or a combination of the first two. Upon pre-treatment for 30 min with each of the compounds no change of impedance was detected (data not shown). As observed in Fig. 2D, pre-treatment with NBTI and the consecutive treatment with adenosine led to an increased AUC (219 ± 7.3%) (Fig. 3; Table 1). In contrast, pre-treatment with CGS 15943 followed by adenosine stimulation resulted in a cellular response with a strongly diminished AUC (29.4 ± 9%) (Fig. 3). Interestingly, when cells were simultaneously pre-treated with CGS 15943 and NBTI, an AUC similar to pre-treatment with CGS 15943 alone was observed (42.2 ± 16%) (Fig. 3). This result indicates that the increased extracellular concentration of adenosine, caused by NBTI inhibition of ENT1, cannot generate an increased cellular response when ARs are blocked by CGS 15943. Similarly, pre-treatment of cells with PSB 1115 resulted in a decreased AUC (18.5 ± 5.4%) (Fig. 3), indicating the A_2B_ AR to be the most prevalent AR. Taken together, this shows that inhibition of ENT1 results in an increased apparent potency for adenosine-mediated AR signalling.

### Pharmacological characterization of ENT1 inhibitors in U-2 OS cells using the xCELLigence

**Figure 4:**
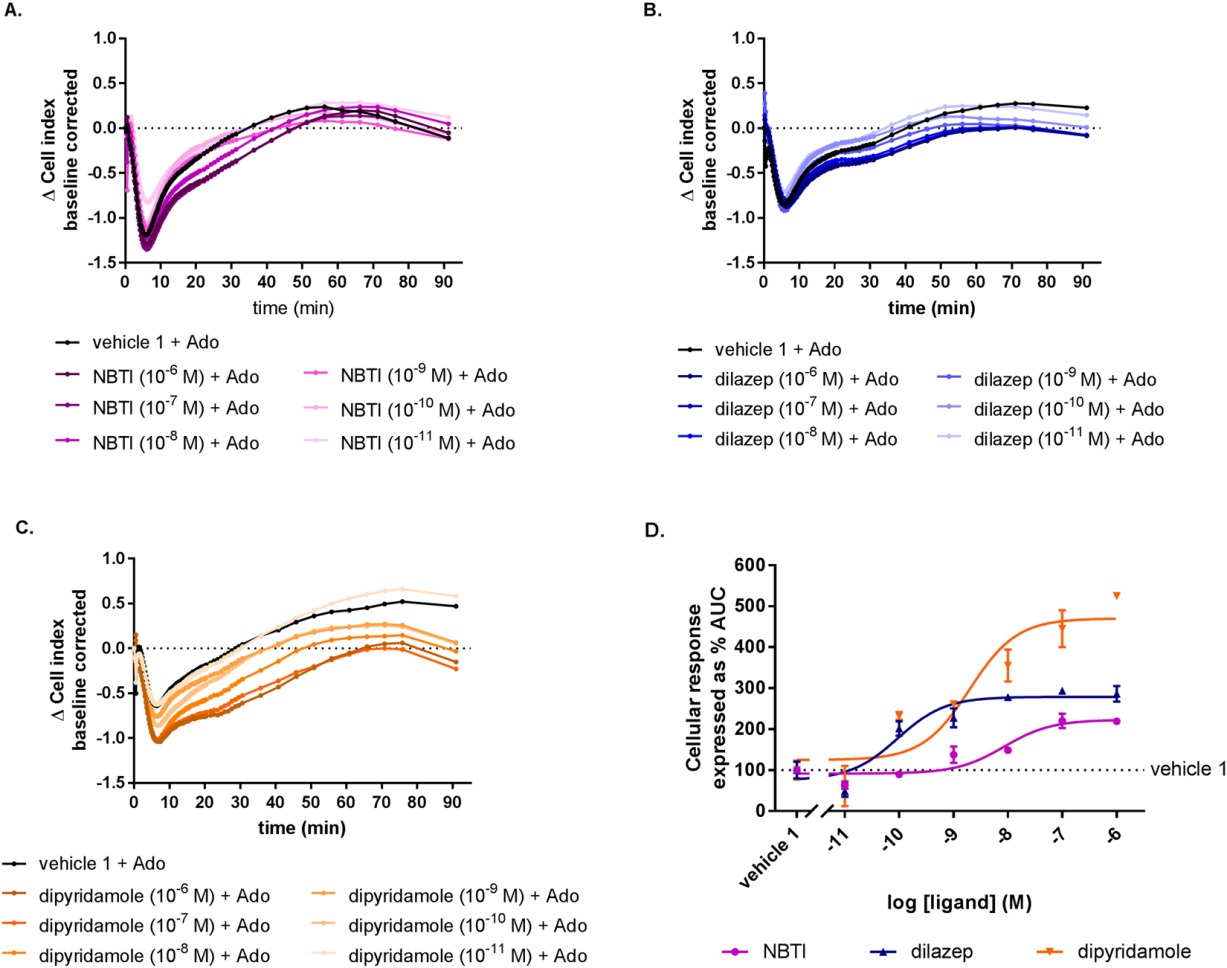
Concentration-dependent effects of ENT1 inhibitors after subsequent AR stimulation with a single adenosine concentration (“Format 2”). A representative adenosine response (10^−4.5^ M) after pre-treatment with ENT1 inhibitors, i.e. (**A**) NBTI, (**B**) dilazep and (**C**) dipyridamole. (**D**) Concentration-response curves of ENT1 inhibitors (normalized to the maximal response of adenosine alone (10^−4.5^ M, 100%)). Data shown are mean ± SEM from at least three separate experiments performed in duplicate.

Lastly, the inhibitory effect of different known ENT1 inhibitors was evaluated in a different assay set up (i.e. “Format 2”). Cells were pre-treated with increasing concentrations of NBTI, dilazep and dipyridamole ranging from 10^−11^ to 10^−6^ M. Successive addition of a fixed concentration of adenosine (30 μM) led to a negative response with a peak around 8 to 10 minutes depending on the concentration of the inhibitor. This phase was followed by an increase of the response that in all cases reached plateau within 90 minutes after the adenosine addition (Fig. 4A–C). Of note, the shape of these traces were similar for all experimental set-ups, indicative for a typical signature of adenosine-mediated AR signalling in U-2 OS cells. Moreover, all compounds appeared to enhance the adenosine-mediated AR signalling in a concentration-dependent manner as higher concentrations of the inhibitors seem to decrease further the ΔCI especially after the initial negative peak is reached. Interestingly, pre-treatment with various concentrations of dipyridamole generated a negative response with a significantly higher inhibitory efficacy (% AUC at 1 µM) compared to dilazep and NBTI. In conclusion, using this assay set up an ENT1 inhibitor’s potency and inhibitory efficacy could be obtained. Furthermore, different ENT1 inhibitors have different potencies for indirectly inhibiting adenosine-mediated AR signalling.

## Discussion

The significance of membrane transporters in drug discovery is widely recognized^24^ and their in-depth investigation is essential as they play a vital role both in physiological conditions and in many diseases. Unfortunately, membrane transporters are vastly understudied compared to other groups of target proteins, and the attention they are getting from the scientific community is limited and not indicative of their biological relevance^25^. One of the main reasons is that the available assays to study transporter activity have limitations, as already introduced above. For another large membrane-bound protein family, GPCRs, label-free technologies are nowadays frequently used to assess receptor pharmacology^26–28^. Specifically, it has been shown that agonist-induced adenosine receptor signalling causes cell morphology changes that can be monitored via both optical- and impedance-based label-free assays^29–32^. In addition, many efforts to study ion channels with label-free technologies are reported. Typical examples are the monitor of TRP ion channel activity with an impedance based assay^33^, as well as the study of GIRK channel and GABA_A_ receptor with an optical biosensor^34,35^. Of note, as far as membrane transporters are concerned, there is currently a single report in which an optical-based label-free assay (i.e. Epic by Corning) is used to study the electrogenic transporter SLC34A2^2^. However, this method has not been applied to other electrogenic transporters so far, and still leaves a need for the many non-electrogenic transporters. We therefore established an impedance-based assay suitable for investigating the inhibition of non-electrogenic membrane transporters via the indirect measurement of GPCR-mediated cell signalling on cells that, in this case, endogenously express both targets.

Initial experiments with U-2 OS cells demonstrated that U-2 OS cell line can be used for xCELLigence experiments as they strongly adhere to the bottom of the wells, which is a requirement for impedance measurements^36^. This resulted in a CI, and thus provided a good window of detection for the assay (Fig. 2B). Additionally, radioligand binding assays validated the presence of ENT1 on U-2 OS cell membranes, since, among others, the determined affinity for all ENT1 inhibitors was found to be in good agreement with available literature data^37,38^. The presence of ARs on U-2 OS cells has already been reported previously, hence it was not re-examined^39,40^.

As adenosine is the endogenous substrate and agonist of ENT1 and ARs, respectively, it was chosen as the compound for assay development. Concentration-response curves of adenosine to cells pre-treated with or without a single concentration of ENT1 inhibitors yielded significant differences on the apparent pEC_50_ value of adenosine (Fig. 2, Table 1). The low pEC_50_ value of adenosine measured in the absence of ENT1 inhibitor (4.2 ± 0.1), might indicate that mainly A_2B_AR is expressed in this cell line, as potency values for the other ARs are reported to be at least 100-fold higher^41^. This hypothesis is compatible with the data publically available from “The Human Protein Atlas” showing that A_2B_ is the most prevalently expressed AR subtype on U-2 OS cells^42–45^. Moreover, pre-treatment of cells with the non-selective ARs antagonist CGS 15943^46^, led to a large decrease of subsequent adenosine signalling, evidence that an AR-specific signal had been captured (Fig. 3), while pre-treatment with the selective A_2B_ AR antagonist PSB 1115, resulted in a further decrease of adenosine signalling, confirming the hypothesis of A_2B_ AR being the main player in the signalling pathway. It is important to note that intracellular adenosine concentrations are strictly regulated to be kept low^47,48^. Specifically, adenosine kinase (ADK), is the principle enzyme to regulate intracellular adenosine and metabolise it to adenosine monophosphate (AMP), which could be further phosphorylated to adenosine diphosphate (ADP) and triphosphate (ATP). Consequently, the intracellular and extracellular adenosine concentrations will not be equilibrated, leading to a fast and continuous adenosine transportation inside the cell by ENT1. As a result, the extracellular adenosine concentration are significantly higher when an ENT1 inhibitor is present.

Subsequent experiments established that the current assay allowed the inhibitory effect of ENT1 inhibitors to be measured in two different assay formats: (1) pre-treatment with a fixed concentration of ENT1 inhibitors and subsequent addition of different concentrations of adenosine (Fig. 2) and (2) pre-treatment with different concentrations of ENT1 inhibitors and subsequent addition of a single concentration of adenosine (Fig. 4). In case of the first format, apparent pEC_50_ values were obtained for adenosine in the absence and presence of different inhibitors, while in the second format the changes of the apparent pEC_50_ values are caused by a concentration-dependent effect of ENT1 inhibitors, which thus are best quantified as pIC_50_ values for the inhibitors. For NBTI, a selective inhibitor of ENT1, a potency value (8.3 ± 0.3) comparable to the radioligand binding data (8.7 ± 0.02) (Supplementary Material; Table S1) as well as previously reported data was found^49^. Dilazep appeared to be approximately 10-fold more potent compared to the literature^49^ and the radioligand binding data, which may be explained by the fact that dilazep is a not selective ENT1 and ENT2 inhibitor^50^. The second format also provides information on the inhibitory efficacy of ENT1 inhibitors (i.e. the efficacy of adenosine signalling via AR that results from ENT1 inhibition) on adenosine-mediated receptor signalling (Fig. 4D). Here it was found that dipyridamole had the highest efficacy, which might be explained by the fact that dipyridamole is a non-selective ENT inhibitor, i.e. it has been shown to have high affinity to ENT1, ENT2, ENT3 transporters^51^ and lower affinity to ENT4^52^ as well. Together, this could lead to significantly higher extracellular concentrations of adenosine that explain dipyridamole’s increased inhibitory efficacy compared to an ENT1 selective inhibitor, such as NBTI. In addition, dilazep seems to follow the same trend of increased inhibitory efficacy as it is also a non-selective inhibitors for ENT1. Such hypothesis is reinforced by the fact that it has been shown that all ENTs are endogenously expressed on U-2 OS cells^53–56^. The inhibition of ENT2, ENT3 and ENT4 on U-2 OS cells using this novel method merits further investigation.

## Conclusions

In conclusion, we developed and validated an assay to detect SLC activity via GPCR signalling in living cells, using a label-free whole cell impedance-based system (xCELLigence), which is to our knowledge the first case where a biosensor technology is used to study the activity of inhibitors for non-electrogenic membrane transporters. As a proof-of-concept the inhibition of ENT1 via subsequent AR signalling in human osteosarcoma cells (U-2 OS) that endogenously express these targets, was studied. We were able to show that its inhibition could be monitored sensitively and quantified accurately via the indirect measurement of adenosine-mediated ARs signalling. Thus, this approach offers the possibility to study membrane transporters that are or can be linked to a GPCR via their common substrate/ligand. Ultimately, this label-free whole-cell assay technology opens novel opportunities for membrane transporter drug discovery.

## Methods

### Materials and reagents

Dipyridamole was kindly provided by Janssen Pharmaceutics and dilazep was obtained from Asta-Werke (Degussa Pharma Gruppe, Bielefeld, Germany). NBTI and adenosine were purchased from Sigma-Aldrich (St. Louis, MO, USA), while CGS 15943 was purchased from Tocris Bioscience (Bristol, UK). PET E-plates 16 and 96 for the xCELLigence DP and SP system (ACEA Biosciences, San Diego, CA, USA) were obtained from Bioké (Leiden, the Netherlands). Homo sapiens bone osteosarcoma cells (U-2 OS) were a kind gift from Mr. Hans den Dulk (Leiden Institute of Chemistry, department of Molecular Physiology, Leiden University, the Netherlands). All other compounds and materials were obtained from standard commercial sources.

### Cell culture

U-2 OS cells were cultured in Dulbecco’s Modified Eagle’s Medium (DMEM) supplemented with stable glutamine 10% (v/v) New Born Calf Serum (NBCS), 100 IU/ml penicillin and 100 mg/ml streptomycin at 37 °C and 7% CO_2_. Cells were cultured as a monolayer on 10 cm ø plates and used for whole cell experiments when a confluency of ~90% was reached.

### Membrane preparation

U-2 OS cells were grown as a monolayer in 15 cm ø plates to 80–90% confluency. Then they were detached by scraping into 5 ml of phosphate-buffered saline (PBS) and subsequently centrifuged for 5 min at 1500 rpm to remove PBS. The pellets were resuspended in ice-cold 50 mM Tris-HCl buffer, pH 7.4 and homogenized with an Ultra Turrax homogenizer (IKA-Werke GmbH & Co.KG, Staufen, Germany). Membranes and the cytosolic fraction were separated by centrifugation at 31,000 rpm in an Optima LE-80 K ultracentrifuge (Beckman Coulter, Fullerton, CA) at 4 °C for 20 min. The pellet was resuspended in 10 mL of Tris-HCl buffer and the homogenization and centrifugation step were repeated. Finally the membrane pellet was resuspended in 50 mM Tris-HCl buffer, pH 7.4, and aliquots were stored at −80 °C. Membrane protein concentrations were measured using a BCA protein determination^57^.

### Radioligand binding assay

U-2 OS membranes were thawed, homogenized using an Ultra Turrax homogenizer at 24,000 rpm (IKA-Werke GmbH & Co.KG, Staufen, Germany) and diluted to the desired concentration (6 μg/well) using assay buffer (50 mM Tris-HCl pH 7.4). Displacement experiments were performed using 10 concentrations of competing ligand in the presence of a final concentration of 4 nM [^3^H]NBTI. At this concentration, total binding (TB) did not exceeded 10% of the radioligand present in the assay in order to prevent ligand depletion. Nonspecific (NSB) binding was determined in the presence of 10^−5^ M NBTI. Total reaction volume was 100 μL and final concentrations of DMSO were ≤0.25%. The experiment was initiated by addition of membranes. Samples were incubated at 25 °C for 60 min to reach equilibrium. The incubation was terminated by rapid vacuum filtration over GF/C filter using Brandel Harvester 24w (Brandel, Gaithersburg, MD, USA). Filters were subsequently washed three times using ice-cold wash buffer (50 mM Tris-HCl, pH 7.4). After drying the filters at 55 °C for 30 min, filter-bound radioactivity was determined by liquid scintillation spectrometry using a Tri-Carb liquid scintillation counter (PerkinElmer, Groningen, The Netherlands).

### Label-free whole-cell assay

#### Detection principle

Label-free whole-cell assays were performed using the xCELLigence real-time cell analyser (RTCA) system^8,9^, as described previously^26^. In short, the RTCA system measures the electrical impedance generated by cells adhering to gold-coated electrodes embedded on the bottom of microelectronic E-plates. Variations in number, degree of adhesion, cellular viability and morphology of cells result in relative changes in impedance (Z), which are recorded continuously at 10 kHz and displayed in the unitless parameter coined Cell Index (CI)^7,8^. CI is a relative measure defined as (Z_i_-Z_0_) Ω/15 Ω, where Z_i_ is the impedance at each individual time point and Z_0_ represents the baseline impedance in the absence of cells, which is measured prior to the start of the experiment.

#### General Protocol

U-2 OS cells were harvested by re-suspending in cell culture medium after brief trypsinization (treatment with trypsin/EDTA for about 5 min) and centrifuged once at 200 × *g* (1500 rpm) for 5 min. Background impedance (Z_0_) was measured after the addition of 40 μL culture media to 16 or 96 well E-plates. Cells were seeded by adding 50 μL of cell suspension containing 20,000 cells per well. After resting at room temperature for at least 30 min, the E-plate was placed into the recording station situated in a 37 °C and 5% CO_2_ incubator. Impedance was measured every 15 minutes overnight.

Cell pre-treatment: Cells were pre-treated by an ENT1 inhibitor (10^−6^ M or 10^−6^ to 10^−11^ M, depending on the assay), adenosine receptor antagonist or vehicle control (0.25% dimethylsulfoxide (DMSO) in Phosphate-buffered saline (PBS)) in 5 µl after 18 h. CI was recorded for at least 30 minutes with a recording schedule of 15 second intervals for 20 minutes, followed by intervals of 1 minute.

Cell treatment: Afterwards, cells were stimulated with adenosine (10^−4.5^ or 10^−6^ to 10^−3.5^ M depending on the assay) or vehicle control (0.125% DMSO in PBS) in 5 μL and CI was recorded for at least 90 minutes with a recording schedule of 15 second intervals for 20 minutes, followed by intervals of 1 minute for 10 minutes and finally 5 minutes. In all cases, final well volumes and DMSO concentrations upon cell and ligands addition were 100 μL and 0.375%, respectively, for all wells and assays.

### Data analysis

#### Radioligand binding assay

Data analyses were performed using GraphPad Prism 7.0 software (GraphPad Software Inc., San Diego, CA, USA). pIC_50_ values in radioligand displacement assays were obtained by non-linear regression curve fitting into a sigmoidal concentration-response curve using the equation:

$${\rm{Y}}={\rm{Bottom}}+({\rm{Top}}-{\rm{Bottom}})/({\rm{1}}+{{\rm{10}}}^{\wedge }(X-\mathrm{LogIC50})).$$

pK_i_ values were acquired from pIC_50_ values using the Cheng-Prusoff equation^58^:

$${{\rm{K}}}_{{\rm{i}}}={{\rm{IC}}}_{{\rm{50}}}/(1+[{\rm{radioligand}}]{/{\rm{K}}}_{{\rm{D}}})$$

The K_D_ value of 1.89 nM for [^3^H] NBTI, was obtained by fitting the data from homologous displacement experiments (Supplementary Material; Fig. S1) to the model “One site – Homologous”.

#### Label-free whole-cell assay

RTCA software 2.0 (ACEA Biosciences, Inc.) was used to obtain the experimental data. All data were analysed using GraphPad Prism 7.00 (GraphPad software, San Diego, CA, USA). After subtracting baseline (vehicle control), ligand responses were normalized at the time of ligand addition to obtain Δ Cell Index (Δ CI) values to correct for ligand-independent responses. Cells that were not pre-treated were used as control (vehicle 1) for the ENT1 inhibitor addition, while cells that were not treated with adenosine (vehicle 2) were used as control for the adenosine addition. The time of normalization was either 18 h or 18 h 30 min after cell seeding for analysis of ENT1 inhibitor/ARs antagonist or adenosine effects, respectively.

The absolute values of Total Area Under the Curve (AUC) up to 90 min after adenosine addition were exported to Graphpad Prism for further analysis yielding bar graphs or concentration–response curves. pEC_50_ values of adenosine after pre-treatment of cells with one concentration of ENT1 inhibitors (format 1; Table 1) and pIC_50_ values of ENT1 inhibitors (format 2; Table 1) were obtained using non-linear regression curve fitting of Total AUC data into a sigmoidal dose-response curve with variable slope. Data shown are the mean ± SEM of at least three individual experiments performed in duplicate.

Statistical significance was determined using one-way ANOVA, followed by a Tukey’s post test for comparison of all columns or a Dunnett’s post test when comparing to vehicle. If p-values were below 0.05, observed differences were considered statistically significant.

## Supplementary information

Supplementary info

## Data Availability

The datasets generated during and/or analysed during the current study are available from the corresponding author on reasonable request.
