# Supplementary material for: Label-free detection of transporter activity via GPCR signalling in living cells: A case for SLC29A1, the equilibrative nucleoside transporter 1
Source: Sci Rep. 2019 Sep 24;9:13802. doi: 10.1038/s41598-019-48829-3 (PMC6760145; doi:10.1038/s41598-019-48829-3)
Supplement: Supplementary file 1 — Supplementary info [file 41598_2019_48829_MOESM1_ESM.docx]

**Label-free detection of transporter activity via GPCR signalling in living cells: A case for SLC29A1, the equilibrative nucleoside transporter 1.**

Anna Vlachodimou, Adriaan P IJzerman & Laura H Heitman*

Division of Drug Discovery and Safety, Leiden Academic Centre for Drug Research (LACDR), Leiden University, P.O. Box 9502, 2300 RA Leiden, The Netherlands

**Email Addresses authors**

| Anna Vlachodimou | a.vlachodimou@lacdr.leidenuniv.nl |
| --- | --- |
| Adriaan P IJzerman | ijzerman@lacdr.leidenuniv.nl |
| Laura H Heitman | l.h.heitman@lacdr.leidenuniv.nl |

***Corresponding author:** Laura H Heitman**,** Division of Drug Discovery and Safety, Leiden Academic Centre for Drug Research, Leiden University, P.O. Box 9502, 2300 RA Leiden, The Netherlands. Tel: +31 71 527 4558. Email: [l.h.heitman@lacdr.leidenuniv.nl](mailto:l.h.heitman@lacdr.leidenuniv.nl)

**Supplementary** **material**

**S1:**


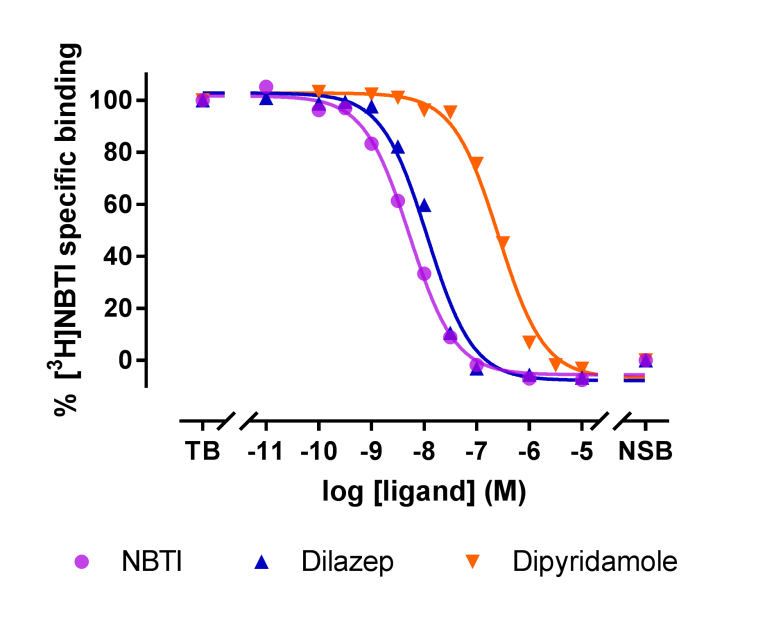


**Figure S1:** Displacement curves of [^3^H]NBTI by non-labeled NBTI, dilazep and dipyridamole from hENT1 endogenously expressed on U-2 OS membranes. Representative graphs from one experiment performed in duplicate.

**Table S1**

**Affinity values of ENT1 inhibitors obtained from [^3^H]NBTI displacement experiments at U-2 OS membranes.**

|  | **K_D_ (nM)**  **(by homologous displacement)** | **pKi ± SEM**  **(Ki (nM))** |
| --- | --- | --- |
| **NBTI** | 1.9 ± 0.3 | 8.7 ± 0.02  (1.9) |
| **Dilazep** | n.a | 8.5 ± 0.1  (69.6) |
| **Dipyridamole** | n.a | 7.2 ± 0.1  (3.2) |

Values are mean ± SEM of at least three separate experiments performed in duplicate.

n.a. not applicable

**S2:**

**Immunofluorescence staining**

Glass-bottom 96-well imaging plates (Greiner, Product number: 655892) were coated with 10 μg/μl fibronectin (Sigma-Aldrich) and directly used for cell plating. Different densities of cells (from 5.000 till 30.000 cells per well) were seeded and allowed overnight (20 h) to attach and stretch. After overnight incubation, cells were incubated with a fixative and permeabilization solution (1% paraformaldehyde + 0.1 Triton X) for 15 min followed by three washes with PBS. After cell permeabilization and blocking with PBS, 0.2% w/v Triton X-100, 0.5% w/v BSA, pH 7.4 (PTB), cells were stained for β-catenin (BD Transduction Laboratories) diluted in PTB (1:500; stock 250 µg/ml). Next, cells were incubated with secondary staining, Alexa488-labeled goat anti-mouse in combination with 2 μg/ml Hoechst 33258 (Sigma-Aldrich) and Rhodamin phalloidin to visualize nuclei and the F-actin cytoskeleton respectively. Cells were imaged using a Nikon Eclipse TE2000-E confocal microscope fitted with a ×20 objective (0.75 NA, 1.00 WD) at 1024x1024 pixels with a final resolution of 0.62 µM/px.


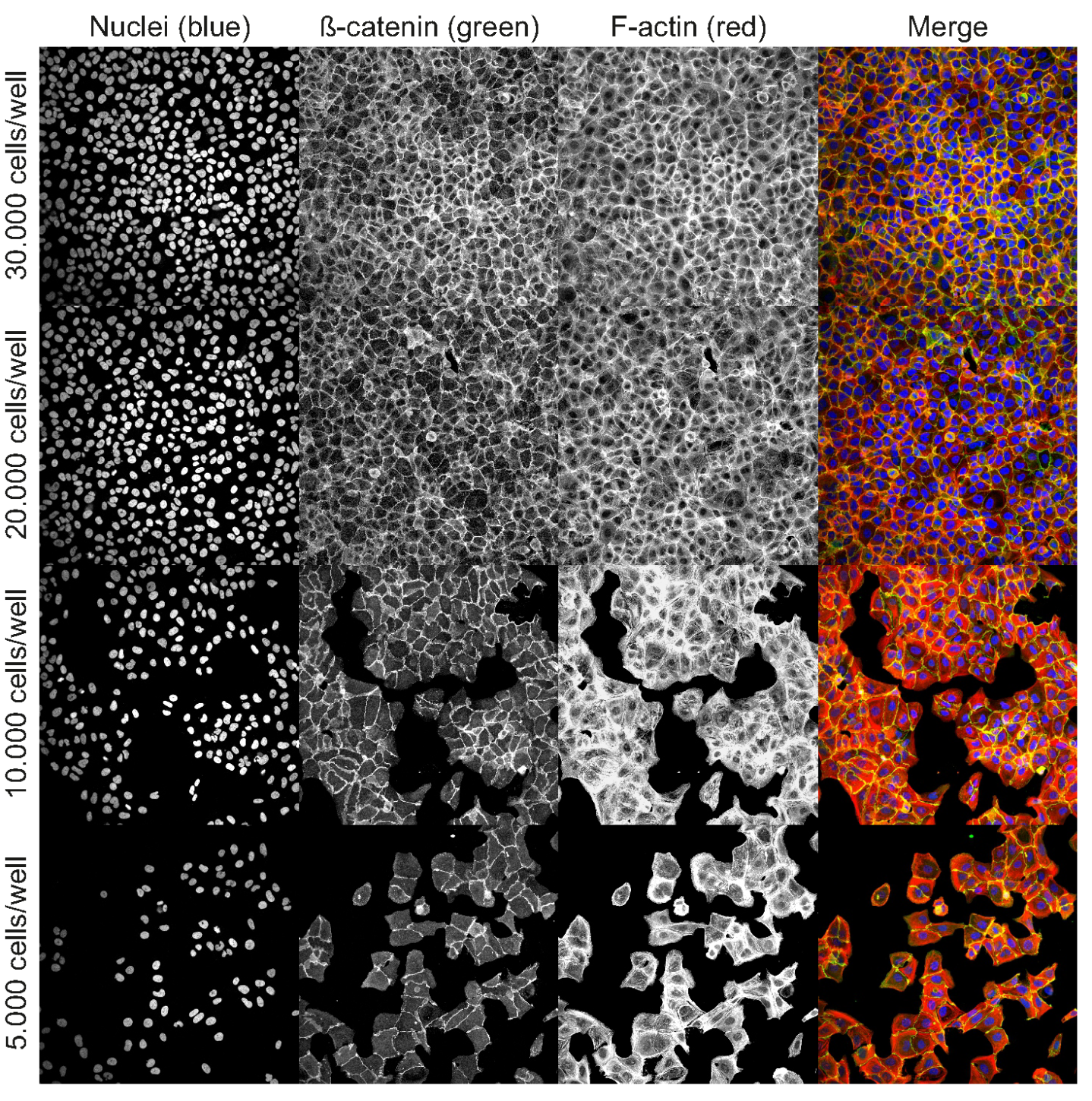


**Figure S2:** Immunofluorescent staining of the cells after 20 h.
